# Fuchs Endothelial Corneal Dystrophy: Strong Association with rs613872 Not Paralleled by Changes in Corneal Endothelial *TCF4* mRNA Level

**DOI:** 10.1155/2015/640234

**Published:** 2015-09-16

**Authors:** Monika Ołdak, Ewelina Ruszkowska, Monika Udziela, Dominika Oziębło, Ewelina Bińczyk, Aneta Ścieżyńska, Rafał Płoski, Jacek P. Szaflik

**Affiliations:** ^1^Department of Genetics, World Hearing Center, Institute of Physiology and Pathology of Hearing, Warsaw, Poland; ^2^Department of Histology and Embryology, Medical University of Warsaw, Warsaw, Poland; ^3^Postgraduate School of Molecular Medicine, Medical University of Warsaw, Warsaw, Poland; ^4^Department of Ophthalmology, Medical University of Warsaw, Warsaw, Poland; ^5^Department of Medical Genetics, Medical University of Warsaw, Warsaw, Poland

## Abstract

Fuchs endothelial corneal dystrophy (FECD) is a common corneal endotheliopathy with a complex and heterogeneous genetic background. Different variants in the *TCF4* gene have been strongly associated with the development of FECD. *TCF4* encodes the E2-2 transcription factor but the link between the strong susceptibility locus and disease mechanism remains elusive. Here, we confirm a strong positive association between *TCF4* single nucleotide polymorphism rs613872 and FECD in Polish patients (OR = 12.95, 95% CI: 8.63–19.42, *χ* ^2^ = 189.5, *p* < 0.0001). We show that *TCF4* expression at the mRNA level in corneal endothelium (*n* = 63) does not differ significantly between individuals with a particular *TCF4* genotype. It is also not altered in FECD patients as compared to control samples. The data suggest that changes in the transcript level containing constitutive *TCF4* exon encoding the amino-terminal part of the protein seem not to contribute to disease pathogenesis. However, considering the strong association of *TCF4* allelic variants with FECD, genotyping of *TCF4* risk alleles may be important in the clinical practice.

## 1. Introduction

Fuchs endothelial corneal dystrophy (FECD) affects approximately 4% of the population over the age of 40 and is the most common genetic disorder of the corneal endothelium. The disease usually has a late onset and presents clinically during the fifth or sixth decade of life [1]. It is characterized by thickening of Descemet's membrane and deposition of extracellular matrix in the form of guttae [2]. Patients with FECD have a reduced density of corneal endothelial cells. As the cells regulate corneal hydration and maintain its transparency, their loss may eventually progress to corneal oedema and vision loss [1]. For the end-stage disease transplant surgery represents the only definitive treatment. FECD is a leading indication for corneal transplantation and Descemet's Stripping Automated Endothelial Keratoplasty (DSAEK) is considered a standard procedure for these patients [3].

Genetic basis for FECD is complex and heterogeneous. Early-onset form of endothelial dystrophy with some of the phenotypic features of FECD occurs rarely and displays an autosomal dominant mode of inheritance with mutations in the* COL8A2* gene (1p34.3, MIM^*∗*^120252). The more common late-onset FECD begins usually after the age of 40 years and both familial and sporadic FECD cases have been described. Familial late-onset FECD is inherited in autosomal dominant fashion. However, the majority of late-onset FECD cases are sporadic with a negative family history. It is much more common and severe in females than in males (3-4 : 1) [4], which contradicts a strictly autosomal dominant transmission but follows multifactorial inheritance. Higher risk of cornea guttata was independently associated with older age, female sex, and a thinner central corneal thickness [5] as well as genetic variants [6].

To date, four different genes* ZEB1* (10p11.22, MIM^*∗*^189909),* AGBL1* (15q25.3, MIM^*∗*^615496),* LOXHD1* (18q21.1, MIM^*∗*^613072), and* SLC4A11* (20p13, MIM^*∗*^610206) as well as four causal loci on chromosomes 5q33.1–q35.2 (FCD3), 9p22.1–p24.1 (FCD4), 13pter-q12.13 (FCD1), and 18q21.2–q21.32 (FCD2), together with a number of susceptibility loci, have been implicated in the pathogenesis of FECD (summarized in Table 1) [7, 8]. A strong association has been established between* TCF4* gene (18q21.2, MIM^*∗*^602272) variants and FECD. A genome-wide association study has identified a common biallelic deep intronic* TCF4* single nucleotide polymorphism (SNP, rs613872; NG_011716.1:g.50559C>A) as a highly significant risk factor for FECD [6]. In addition to that, a separate study reported a trinucleotide expansion (CTG18.1) in the intron region of* TCF4,* which is highly prevalent in FECD individuals [9]. The role of these associations in FECD development remains poorly understood. In the study we set out to analyze whether the presence of* TCF4* rs613872 risk allele affects the expression of* TCF4* in corneal endothelial cells, which would provide a functional link between a risk factor and disease mechanism in FECD.

## 2. Patients and Methods

**Figure 1:**
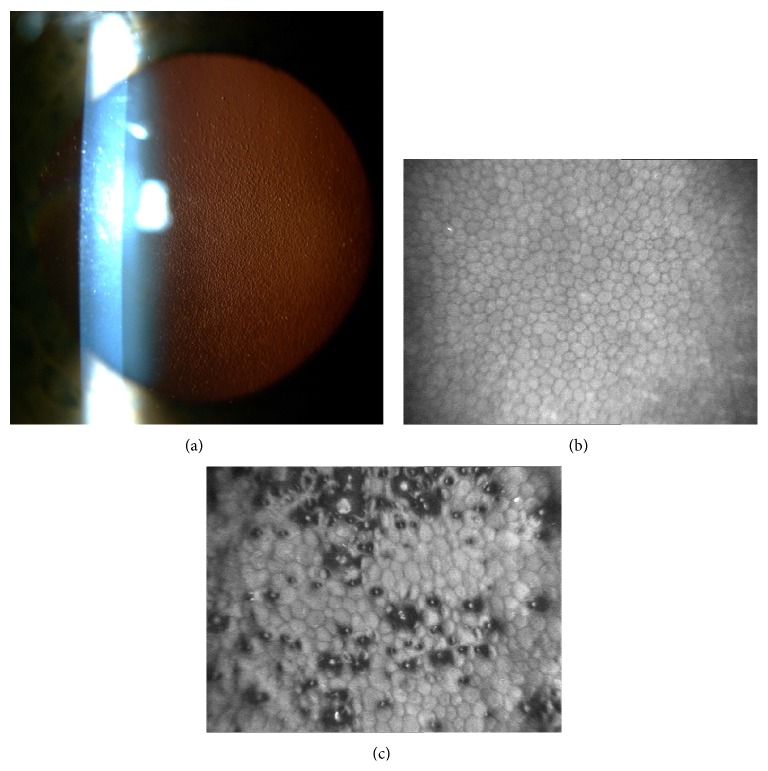
Characteristic features of FECD. (a) Slit-lamp photography shows the presence of pathological guttae, focal excrescences of Descemet's membrane at the level of corneal endothelium in a patient with FECD. (b) Confocal microscopy image of the corneal endothelium in a control subject demonstrates a regular mosaic of the endothelial monolayer with bright cell bodies and dark, hexagonal cell boundaries. (c) Confocal microscopy in a patient with FECD reveals pleomorphism and polymegathism of the endothelium and typical guttae as dark bodies with a central bright reflex.

The study conformed to the tenets of the Declaration of Helsinki and was approved by the local Ethics Committee. Patients were recruited from the Department of Ophthalmology, Medical University of Warsaw, and gave informed consent prior to participation. The diagnosis of FECD was based on the visualization of “guttae” and stromal oedema by slit-lamp examination, confocal microscopy* in vivo* (IVCM, Confoscan 4, Nidek Technologies), and anterior segment optical coherence tomography (CASIA Cornea/Anterior Segment OCT SS-1000, Tomey) (Figure 1).

### 2.1. Genotyping

Genomic DNA was isolated from peripheral blood samples of sporadic, unrelated FECD patients (*n* = 252; 187 females and 65 males) with a standard salting-out procedure. Control DNA samples (*n* = 323), representative of the background population of central Poland, came from the repository of the Department of Medical Genetics [10]. The* TCF4* rs613872 was genotyped using ABI Custom TaqMan SNP Genotyping Assay (Applied Biosystems) and the real-time PCR system (Viia7, Thermo Fisher Scientific). The accuracy of genotyping was confirmed by Sanger sequencing in selected subjects.

### 2.2. RNA Isolation and Quantitative Real-Time PCR

Corneal endothelial cell monolayers attached to Descemet's membrane were obtained from FECD patients (*n* = 40) during endothelial keratoplasty (DSAEK) or excised from donor corneoscleral buttons (*n* = 23) that were not used for transplantation. The FECD patients and controls included in the RNA studies derived from the group genotyped at rs613872 (FECD group: TT *n* = 3, TG *n* = 31, GG *n* = 6; control group: TT *n* = 14, TG *n* = 6, GG *n* = 3). The specimens were submerged in the RNAlater storage solution (Ambion, Austin, TX, USA) and frozen immediately. Total RNA was extracted using Trizol (Thermo Fisher Scientific, Waltham, MA, USA) and cDNA was generated from 500 ng of RNA with the Maxima First Strand cDNA Synthesis Kit (Thermo Fisher Scientific). Gene expression was measured by real-time PCR (Viia7, Thermo Fisher Scientific) using PCR primers (Oligo, Warsaw, Poland) and probes (Roche Universal Probe Library) designed with the ProbeFinder software, version 2.50 (Roche). The analyzed coding region of* TCF4* is present in both TCF4-A and TCF4-B isoforms. It is located in the amino-terminal part of the protein, close to the activation domain 2 (AD2). For* TCF4* quantitative PCR (qPCR), the primer pair 5′-GCACTTTCCCTAGCTCCTTCT-3′ and 5′-GCATAGCCAGGCTGATTCAT-3′ and probe 25, for* RPL13A* (60S ribosomal protein L13a) qPCR the primer pair 5′-CTGGTGCTTGATGGTCGAG-3′ and 5′-GTTGATGCCTTCACAGCGTA-3′ and probe 77 were used. Expression values for* TCF4* were calculated using the modified double delta Ct (ΔΔCt) method and absolute quantification and normalized to* RPL13A* [11].

### 2.3. Statistical Analysis

Hardy-Weinberg equilibrium (HWE) was analyzed with the *χ* ^2^ test in the patient and control groups. The odds ratios (ORs) with 95% confidence intervals (95% CI) and *p* values were calculated with the Web-Assotest program (http://www.ekstroem.com/assotest/assotest.html). Differences in gene expression between the groups were analyzed with a two-sided unpaired *t*-test (Statistica, StatSoft, Poland). Pearson's correlation was used to assess the degree of the relationship between gene expression and age or sex. *p* < 0.05 was considered to indicate statistical significance.

## 3. Results

Both the patient and control groups were genotyped for the* TCF4* SNP rs613872. In FECD patients the distribution of the TT, TG, and GG genotypes showed a significant deviation from Hardy-Weinberg equilibrium (*χ* ^2^ = 31.1, *p* < 0.0001), while in control subjects the genotype distribution remained in the Hardy-Weinberg equilibrium (*χ* ^2^ = 1.3, *p* = 0.265). Allele G was significantly more prevalent in patients with FECD than among control subjects (Table 2). The odds ratio (OR) for two copies of the risk allele (GG homozygotes versus TT homozygotes) was 20.87 (95% CI: 9.42–46.24), whereas the OR for one copy of the risk allele G (TG heterozygotes versus TT homozygotes) was 11.99 (95% CI: 7.90–18.19). Testing the association between FECD and the* TCF4* genotype under dominant, additive, or recessive models, we found that the most plausible model was the dominant one whereas the additive and recessive models could be formally rejected as indicated by the *p* values for model fit (*p* < 0.0001). The dominant model (combining the GG and TG into one category) conferred the highest OR of all the tested models (OR = 12.95, 95% CI: 8.63–19.42, *χ* ^2^ = 189.5, *p* < 0.0001).

**Figure 2:**
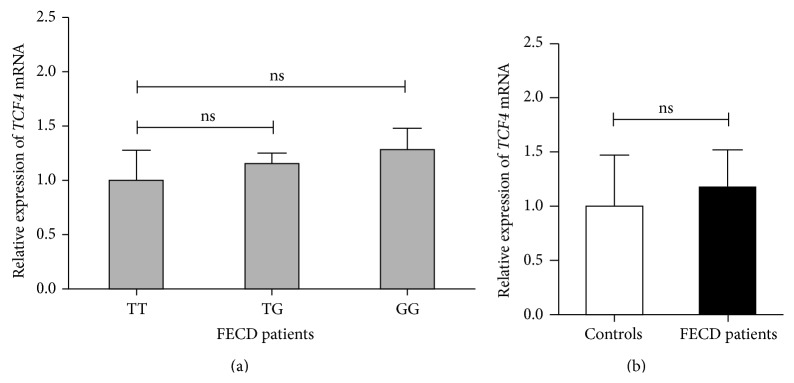
Expression of* TCF4* in corneal endothelial cells. The amount of* TCF4* mRNA was quantified by real-time PCR in relation to RPL13A. (a) Expression of* TCF4* in FECD patients with respect to a different* TCF4* genotype at rs613872 (TT *n* = 3, TG *n* = 31, and GG *n* = 6) is shown; ns: nonsignificant. (b) Average values of* TCF4* mRNA expression in control samples (white bars) and FECD patients (black bars) are shown; ns: nonsignificant.

Expression of* TCF4* was not age or sex dependent. There were no differences in* TCF4* mRNA levels in FECD patients with a particular* TCF4* SNP rs613872 genotype (Figure 2(a)) but also when* TCF4* genotypes of patients and controls were analyzed together (data not shown). No increase in the amount of* TCF4* mRNA transcripts was observed in FECD patients as compared to the control group (Figure 2(b)).

## 4. Discussion

Our data are in accord with previous studies showing that the presence of a* TCF4* risk allele at rs613872 is much more common in Caucasian patients with FECD and strongly predisposes to the development of the corneal dystrophy [6, 12–16]. Indian FECD patients do not show a distinct association with this* TCF4* variant, which may be explained by a small sample size studied [17]. In Chinese FECD patients no association could be observed as the population is not polymorphic at this genomic position [18–20]. In these FECD patients,* TCF4* genetic variants adjacent to rs613872 (e.g., rs17089887 in both Indian and Chinese subjects) were strongly associated with FECD, which suggested the presence of significant disease-causing changes in the nearby regions of these alleles [17, 19, 20]. All the relevant SNPs in the respective populations are in strong linkage disequilibrium with a newly identified CTG18.1 trinucleotide repeat expansion in the* TCF4* gene [9, 17, 20, 21], which confers a transethnic association with FECD.

*TCF4* is now widely recognized as a major contributor to FECD. One may assume that patients carrying* TCF4* risks alleles and thus having an increased susceptibility to FECD should more often have follow-up ophthalmic examinations. Diagnosis of cataract in these patients might be an indication for an earlier cataract surgery before a full-blown, clinically significant FECD develops and the patients require a combined procedure of corneal transplantation and cataract surgery.

*TCF4* genetic variants are strongly associated with FECD but so far little is known about a possible involvement of* TCF4* gene products in the development of FECD. Hypothesizing that the strong genetic association may indicate a causal relationship and the* TCF4* genetic variant may represent a tissue-specific regulatory element,* TCF4* mRNA studies in corneal endothelial cells were performed. Analyzing more than 60 different corneal endothelium and Descemet's membrane complexes, we have not found any significant differences in the expression of* TCF4* at mRNA level in FECD patients as compared to control samples. There were no differences in* TCF4* expression between the carriers and noncarriers of the* TCF4* risk allele neither in the group of FECD patients alone nor when FECD patients and controls were pooled together. The data suggest that changes in the transcript level containing constitutive* TCF4* exon encoding the amino-terminal part of the protein seem not to contribute to disease pathogenesis.

Recently, unaltered* TCF4* expression was also reported in another group of FECD patients [22]. In the absence of distinguished differences in the amount of mRNA, transcript activity still may be altered or posttranslational mechanisms may affect protein structure, level, or cellular distribution. A novel and so far the only identified link between* TCF4* susceptibility and FECD disease mechanism is the formation of toxic poly(CUG)n RNA and missplicing events in patients with* TCF4* repeat expansion. In corneal endothelial cells of these patients the* TCF4* repeats are actively transcribed and seem to preferentially accumulate into poly(CUG) RNA foci. A protein found to be immobilized in RNA foci is MBNL1, a splicing regulator. Consequently, differential splicing events were found in corneal endothelium of FECD patients [23].

Our study confirms and extends the association of* TCF4* with FECD by testing a novel previously not analyzed population. It advances our knowledge on the role of* TCF4* in the development of FECD. Genotyping of* TCF4* risk alleles might be considered as a diagnostic procedure as the genetic results have an important predictive value and are of clinical utility.

## Figures and Tables

**Table 1:** Loci, genes, and genetic variants related to late-onset FECD.

Chromosome	Locus/gene/variant	Late-onset FECD
1p35.1	rs760594	F
5q12.3	rs1301475	F
5q33.1–q35.2	FCD3	F
7q22.3	rs257376	F
8p21.3	rs2466216	F
8p21.1	rs9797	F
8q21.13	rs1380229	F
9p22.1–p24.1	FCD4	F
10p11.22	*ZEB1 *	F/S
10q24.2	rs1889974	F
13pter-q12.13	FCD1	F
15q22.2	rs235512	F
15q22.31	rs352476	F
15q25.3	*AGBL1 *	F/S
17q25.3	rs938350	F
18q21.1	*LOXHD1 *	F/S
18q21.2	*TCF4 *	F/S
18q21.2–q21.32	FCD2	F
20p13	*SLC4A11 *	F/S
20p12.2	rs674630	F
Xq28	rs1990383	F

F: familial, S: sporadic.

**Table 2:** Genotype distribution and allele frequency of *TCF4* rs613872 in patients with FECD and control subjects.

*TCF4* rs613872 genotype	Genotype counts, *n* (%)				OR dominant model (95% CI)	Allele counts, *n* (%)		*χ* ^2^ statistic, *p*
	TT	TG	GG	Total		T	G	
FECD patients	46 (18.25%)	170 (67.46%)	36 (14.29%)	252	12.95 (8.63–19.42)	262 (51.98%)	242 (48.02%)	156.7 <0.0001
Controls	240 (74.30%)	74 (22.91%)	9 (2.79%)	323		554 (85.76%)	92 (14.24%)	
